# Prospective Associations Among Loneliness and Health for Servicemembers: Perceived Helplessness and Negative Coping Appraisal as Explanatory Mechanisms

**DOI:** 10.3390/bs15091240

**Published:** 2025-09-11

**Authors:** Sarah N. Arpin, Cynthia D. Mohr, Todd E. Bodner, Leslie B. Hammer, James D. Lee

**Affiliations:** 1 Department of Psychology, Gonzaga University, Spokane, WA 99258, USA; 2 Department of Psychology, Portland State University, Portland, OR 97201, USA; cdmohr@pdx.edu (C.D.M.); tbodner@pdx.edu (T.E.B.); hammerl@ohsu.edu (L.B.H.); 3 Oregon Institute of Occupational Health Sciences, Oregon Health and Sciences University, Portland, OR 9723, USA; 4 Walter Reed Army Institute of Research, Silver Spring, MD 20910, USA; james.d.lee208.mil@health.mil

**Keywords:** loneliness, stress, sleep, alcohol use, military health

## Abstract

Links between loneliness and health are robust, though evidence for associations with alcohol use is mixed. Previous research has supported perceived stress as a predictor of alcohol use and as a pathway through which loneliness impacts health over time. Yet findings are primarily limited to civilian samples, and less is known about how loneliness relates to stress and health among service members. The current study explores prospective associations among loneliness, stress, and health (i.e., sleep, alcohol misuse, and psychological distress) within a sample of mostly male service members. We examine two dimensions of perceived stress, perceived helplessness and negative coping appraisal, as explanatory mechanisms. Controlling for baseline stress and health, loneliness predicted perceived helplessness and negative coping appraisal (4-month follow-up); in turn, perceived helplessness and negative coping appraisal predicted insomnia and sleep dissatisfaction; and negative coping appraisal predicted alcohol misuse (indirect effects). Findings support transactional models of stress and the stressor-vulnerability model of alcohol use, revealing that coping appraisals play an important explanatory role for stress-related consequences of loneliness. Further, we provide new insight into mechanisms linking loneliness to alcohol use and sleep, differentiating dimensions of stress and highlighting potential intervention targets.

## 1. Introduction

Loneliness is a distressing emotional experience, linked to physiological and psychological decline (Hawkley & Cacioppo, 2010). Defined as the perceived discrepancy between desired and actual social relationships, loneliness is distinct from the objective experience of social isolation (Russell, 1982). Perceived isolation is an aversive experience, and much like the experience of physical pain, is thought to signal a problem (i.e., lack of social connection) and motivate behavior to reduce or alleviate the source of discomfort (J. T. Cacioppo et al., 2006a). Thus, loneliness evolved as an intensely stressful experience signaling a threat to the “social body” and thus compromised evolutionary fitness (J. T. Cacioppo & Cacioppo, 2018, p. 137). In modern life, the experience of loneliness continues to be aversive, and increases in loneliness are associated with changes in various psychosocial factors, such as depression, hostility, and perceived stress (Hawkley et al., 2006; Ernst & Cacioppo, 1999). Because sustained stress contributes to wear and tear on the body, theory suggests that cumulative stress is a major avenue through which loneliness operates to impact health over time (e.g., J. T. Cacioppo & Cacioppo, 2018; Hawkley et al., 2003).

A recent Surgeon General Advisory warns of the grave impact of increasing rates of social isolation and loneliness, both in the United States and abroad (Office of the Surgeon General, 2023). The report declares a public health emergency, urging scholars and practitioners to address these increasing issues through empirical research, particularly among at-risk groups. The current study responds to this call by investigating prospective associations among loneliness and health outcomes, specifically alcohol use, sleep, and psychological distress. Importantly, we explore perceived stress as an explanatory mechanism in a sample of military service members, who are at a heightened risk for experiencing loneliness, stress, and health-related consequences (e.g., Straus et al., 2022; Bray et al., 2010; Farhadian et al., 2022; Nichter et al., 2020).

### 1.1. Health Consequences of Loneliness

Sustained feelings of loneliness have been shown to trap individuals in a cycle of negative social cognitions and “self-defeating behaviors” that impede positive social interactions (J. T. Cacioppo et al., 2014, p. 6). Research increasingly shows that chronically viewing one’s social world as a place of potential threat is linked to serious declines in physiological and psychological health. More generally, social stressors such as emotional abuse and childhood trauma have been linked to later health risks such as sleep deficits and problematic alcohol use (Noudali et al., 2022). Loneliness, in particular, is linked to depression (Luo et al., 2012), greater cardiovascular health risk and hypertension (Caspi et al., 2006; Vigorito & Giallauria, 2018), poor immune-system functioning (Kiecolt-Glaser et al., 1984), dementia onset (Holwerda et al., 2014), and mortality (House et al., 1988). Overall, the negative health consequences of loneliness are now known to be comparable to, if not greater than, traditional medical risk factors (e.g., smoking, obesity, physical inactivity, and air pollution; Holt-Lunstad et al., 2015). Additional research, though mixed, has found links among loneliness and health behavior (Hawkley, 2022), including greater adverse health behaviors such as drinking (e.g., Arpin et al., 2015; Tanskanen et al., 2021; Bonin et al., 2000; Wakabayashi et al., 2022) and smoking (e.g., DeWall & Pond, 2011; Dyal & Valente, 2015) and reduced health-promoting behavior such as physical activity (e.g., Hawkley et al., 2009; Pels & Kleinert, 2016) and sleep (J. T. Cacioppo et al., 2000). Relatedly, substance use has been described as a common coping response to loneliness (Rokach & Brock, 1998), and more recent research provides preliminary evidence for higher levels of loneliness among substance-dependent populations (Ingram et al., 2020) and individuals with psychiatric and substance use disorders (E. Martin et al., 2023). The consequences of loneliness on substance use were amplified during the COVID-19 pandemic, with some research showing that loneliness predicted increased problematic drinking and drug use in community-dwelling adults (e.g., Horigian et al., 2021; Gutkind et al., 2022; Bragard et al., 2022; Tucker et al., 2022) and among military veterans with pre-existing mental health and substance use issues (Kelly et al., 2022; Fitzke et al., 2021; Reilly et al., 2022).

Importantly, these health and behavioral consequences of loneliness are bidirectional. That is, loneliness predicts poorer health, and chronic health conditions present barriers to social interaction, thus intensifying or prolonging loneliness (Phillips et al., 2023). Indeed, previous work has supported this perspective for multiple health-related consequences of loneliness, including pain (e.g., Suzuki et al., 2023; Loeffler & Steptoe, 2021); objective and subjective health (Phillips et al., 2023); sleep quality (Hawkley et al., 2010a); depression (e.g., Hsueh et al., 2019); and substance use (Wootton et al., 2021). Thus, it is important that research considering causal links among loneliness and health account for potential reverse causality by incorporating prospective, longitudinal designs. For example, though loneliness and depression are strongly related constructs, longitudinal research has found that, in addition to bidirectional associations, loneliness consistently predicts later depression more than depression does loneliness (e.g., Vanhalst et al., 2012). Gutkind et al. (2022) provided important evidence for the longitudinal impact of loneliness on substance use, showing that individuals with moderate to severe levels of loneliness reported greater alcohol and cannabis use at a later time point. Similarly, Wakabayashi et al. (2022) showed that high levels of loneliness predicted an increased likelihood of high-risk drinking and alcohol dependence at a one-year follow-up. Whereas evidence for longitudinal and bidirectional associations among loneliness and health, specifically health behavior, is robust, more work is needed to understand the exact mechanisms through which social pain exerts its influence on health over time (Ong et al., 2016).

### 1.2. Perceived Stress as an Explanatory Mechanism

Even in transient (i.e., temporary) form, loneliness is associated with self-reported psychological stress (J. T. Cacioppo et al., 2000; J. T. Cacioppo et al., 2006a; Vasan et al., 2023); physiological markers of stress including elevated cortisol (Pressman et al., 2005) and higher blood pressure (Hawkley et al., 2006; Hawkley et al., 2010b); and greater stress reactivity (e.g., diastolic blood pressure reactions, Steptoe et al., 2004; stress-related inflammation, Jaremka et al., 2013). The relationship between loneliness and stress is similarly complex as that between loneliness and health, in that it is reciprocal. Loneliness has been shown to be an immediate cause (e.g., J. T. Cacioppo et al., 2006a) but also a consequence of stress (Campagne, 2019). Whereas previous research has established that loneliness and perceived stress co-occur, little research has considered their relationship over time. In a seminal study, Laustsen et al. (2024) explored the longitudinal relationship between loneliness and perceived stress, revealing cross-sectional and bidirectional associations. Specifically, loneliness at baseline predicted greater perceived stress at a four-year follow-up, and perceived stress at baseline similarly predicted later loneliness. Given this bidirectional association, it is important for research to consider how this relationship unfolds overtime. That loneliness and stress are interdependent may provide insight into the persistent and pervasive health outcomes of loneliness.

Indeed, links between loneliness and various health outcomes have been explained by perceived stress. For example, perceived stress mediates associations among loneliness and sleep problems (Griffin et al., 2020); depression (J. T. Cacioppo et al., 2006b; J. C. Martin & Hartley, 2017); and alcohol and drug use (Segrin et al., 2018; Berberian et al., 2022). Research and theory from the more general drinking literature have established stress (and interpersonal stress in particular; Armeli et al., 2007; C. D. Mohr et al., 2001) as an important predictor of alcohol use. Specifically, the tension-reduction model (Conger, 1956) asserts that drinking is used to reduce negative affect caused by stress, given alcohol’s dampening effect on the nervous system. Importantly, this theory supports alcohol use as a coping response to numb social pain (Rokach & Brock, 1998). The stressor-vulnerability model expands on this, proposing that individuals who expect alcohol to have positive effects or who want to avoid dealing with problems are more likely to use alcohol when experiencing stress (Armeli et al., 2000; Armeli et al., 2007). Importantly, whereas the tension reduction model predicts that negative affect increases the likelihood of alcohol use, the stressor-vulnerability model maintains that certain characteristics (e.g., expectancies related to the effects of alcohol, poor coping skills, gender) make some individuals more at risk for using alcohol to cope with stress.

Several hypotheses explaining links among loneliness, stress, and health have also been proposed in the existing loneliness literature. These include the added stress hypothesis, which predicts that how perceptions of loneliness and social rejection are stressors that produce negative affect and subsequently stress-related physiology; the differential-exposure hypothesis, asserting that lonely individuals are exposed to stressful events at greater frequency than nonlonely individuals; the differential-reactivity hypothesis, or that the lonely exhibit more intense responses to stress; and the differential stress-buffering hypothesis predicting lower levels of perceived support among the lonely, and thus fewer resources to support coping with stress (J. T. Cacioppo & Hawkley, 2003). Research has garnered support for all of these hypotheses, with the exception of differential-stress exposure (i.e., the lonely are exposed to stress at a greater frequency). That is, lonely persons are actually exposed to stressors (e.g., daily hassles, major life stressors) at similar levels as the socially connected (e.g., Hawkley et al., 2003; J. T. Cacioppo et al., 2000). However, they report greater negative affect and psychological stress (added stress hypothesis), are more reactive to stress (differential-reactivity), and have fewer support resources available to ameliorate the negative impacts of stress on health (differential stress-buffering). Overall, these hypotheses and the supporting research suggest that lonely persons differ in levels of perceived stress and in their appraisal of coping resources, which, as will be discussed below, are well-known predictors of health outcomes.

### 1.3. Dimensions of Perceived Stress

As articulated by the transactional theory of stress (Lazarus & Folkman, 1984), greater stress occurs when an individual perceives that current stressors exceed coping resources, or when individuals perceive their lives to be unpredictable, uncontrollable, or overloaded (Cohen & Williamson, 1988, p. 34). As such, perceived stress is a transaction between the individual and their environment. Indeed, the most common measure of perceived life stress is the Perceived Stress Scale (PSS; Cohen et al., 1994), which measures the degree to which individuals perceive their life as excessively stressful relative to their ability to cope. This approach falls in line with the differential-reactivity and differential stress-buffering hypotheses for loneliness, which propose links between loneliness, perceived stress, and coping appraisals. The PSS has been shown to reliably assess perceived stress in clinical (Leung et al., 2010; Hewitt et al., 1992), community-dwelling (Cohen & Janicki-Deverts, 2012), and military samples (Davis et al., 2022), with subjects ranging from adolescence to older adulthood (Demkowicz et al., 2020; Taylor, 2015; Ezzati et al., 2014), and is frequently administered in studies of loneliness (e.g., Laustsen et al., 2024; Wang et al., 2024; McHugh & Lawlor, 2013).

While this scale has been traditionally employed as a global measure of perceived stress, more recent research has revealed that all three versions of the measure (i.e., PSS-14, PSS-10, PSS-4) may be multidimensional. Scores may reflect two dimensions of stress, specifically, perceived helplessness or distress, as measured by the negatively-phrased items on the measure (“In the last month, how often have you been unable to control the important things in your life?”), and perceived coping efficacy (referred to as negative coping appraisal in the current study), as measured by the positively-phrased items (“…how often have you felt confident in your ability to handle your personal problems?”). Studies exploring these two interrelated factors have revealed that they have distinct predictive qualities (Taylor, 2015; Demkowicz et al., 2020; Leung et al., 2010). For example, Hewitt et al. (1992) showed that the perceived helplessness dimension of PSS predicted depression in men and women, but that perceived coping ability dimension was predictive of depression in women but not men. Taylor (2015) argued that considering the multidimensional nature of the PSS helps to expand the current understanding of stress to include perceived helplessness and “coping efficacy” as critical, interrelated components. Indeed, research has found that the two dimensions are only moderately correlated (e.g., r = −0.28 to −0.39, Ezzati et al., 2014; Demkowicz et al., 2020), providing further support that these factors represent related but distinct indicators of stress.

Previous research has established a positive correlation between loneliness and global perceived stress, as measured by the PSS (Laustsen et al., 2024). However, no research of which we are aware has examined links between loneliness and the two dimensions of the PSS (perceived helplessness and coping appraisal). As articulated by the loneliness model (Hawkley & Cacioppo, 2010), perceived social isolation is accompanied by a host of psychosocial correlates and cognitive biases, as a result of an increased hypervigilance for social threat in the environment. For example, lonely persons report increased hostility and helplessness (e.g., Loboprabhu et al., 2015) and low perceived control (Solano, 1987), reduced memory of positive social events (Igarashi, 2025), and lower self-esteem (Hu et al., 2013; Ouellet & Joshi, 1986) and self-efficacy (Roskoschinski et al., 2023). Further, lonely people typically exhibit more passive coping strategies such as behavioral disengagement (J. T. Cacioppo et al., 2000) and report lower coping efficacy (J. W. Lee et al., 2023), or one’s belief in his/her ability to cope with stress (Sandler et al., 2000). These consequences set off a loneliness loop as they predict less socially attractive behavior and increased social distance, and thus simultaneously activate mechanisms that are consequential to health over time. Based on the loneliness model and previously documented psychosocial correlates of loneliness, it is predicted that greater loneliness will uniquely relate to the two dimensions of perceived stress, as measured by the Perceived Stress Scale (Cohen et al., 1994). Specifically, greater loneliness will predict greater perceived helplessness and more negative coping appraisal.

There is some evidence to support positive associations between perceived helplessness and coping appraisal with health outcomes explored in the current study (i.e., sleep, alcohol misuse, psychological distress). For example, perceived helplessness predicts sleep problems (Ejdemyr et al., 2021) and alcohol misuse (e.g., Timmer et al., 1985; Castellanos-Perilla et al., 2022). Likewise, helplessness is strongly correlated with psychological distress (e.g., Zautra & Wrabetz, 1991) and plays a role in the development of depression (Broos et al., 2023). As a construct, coping efficacy is related to drinking behavior (e.g., Dyar et al., 2021), psychological distress and depression (Cunningham et al., 2020; Manne & Glassman, 2000), and sleep (Ten Brink et al., 2021). Yet, no research of which we are aware has considered how perceived helplessness and coping appraisals, as dimensions of stress, predict loneliness-related outcomes. Thus, our examination of specific indirect effects and the potentially unique predictive quality of each dimension is exploratory. Examining two dimensions of stress as distinct mechanisms of influence could facilitate more targeted interventions for loneliness and health, thus making this an important avenue of research to explore.

While relationships among loneliness, stress, and health outcomes are nuanced, research has provided clear evidence in support of perceived stress as a mechanism for links among loneliness and health, and in particular for behavioral health outcomes (e.g., substance use, sleep health). Yet, much of this work is limited in that it is either cross-sectional (e.g., Christiansen et al., 2016; Segrin & Passalacqua, 2010), does not adequately control for baseline stress (e.g., Segrin et al., 2018), or focuses primarily on samples of adolescents, college students, or older adults. Given the bidirectional associations among loneliness, stress, and health (e.g., Phillips et al., 2023; Park et al., 2020; Laustsen et al., 2024), it is important for research to consider how loneliness relates to downstream psychological and health consequences, above and beyond cross-sectional and autoregressive associations among these constructs. Further, research must explore these processes in diverse samples to further generalize existing research and theory. To address gaps in existing research, the current study employs a prospective, longitudinal design to explore downstream consequences of loneliness and concomitant stress-related consequences. Importantly, we explore these processes in the unique context of the military, which, as will be discussed below, may present unique experiences and health consequences of loneliness and stress.

### 1.4. The Military as a Cultural Context

Whereas previous research has provided ample evidence for the role of perceived stress in loneliness-health behavior associations, findings are primarily limited to civilian samples. Less is known about how loneliness relates to stress and subsequent health and psychosocial outcomes among service members, who are particularly at risk for loneliness (Wilson et al., 2018; Straus et al., 2022). J. T. Cacioppo et al. (2016) have argued that, while the experience of loneliness is universal, predictors of loneliness are culturally sensitive. The military is one cultural context within which individuals present unique experiences of loneliness and isolation. For example, in a study of active-duty soldiers, J. T. Cacioppo et al. (2016) found that relationship quality with friends and platoon members was a significant buffer against loneliness, whereas relationship quality with family and marital partner (which typically predict less loneliness in civilian populations) were not. A recent study of military veterans revealed that, whereas overall loneliness did not increase during the pandemic, certain risk factors (e.g., isolation, stress) made some service members more vulnerable to increases in loneliness (Na et al., 2022); such increases in loneliness were more in line with trends observed in the general population (Ernst et al., 2022).

Though social isolation and loneliness (at moderate to severe levels) are common experiences among military veterans (e.g., Kuwert et al., 2014; Straus et al., 2022), there is a dearth of research investigating health and behavioral consequences of veteran loneliness. The stigma of disclosing loneliness may prevent lonely servicemembers from seeking support (Hoge et al., 2004; J. T. Cacioppo et al., 2016), thus leading to the maintenance of loneliness and exacerbation of health consequences. Recent evidence supports the utility of workplace interventions targeting supervisory support to reduce loneliness. For instance, C. Mohr et al. (2024) demonstrated that a brief, theory-based supportive leadership training significantly reduced loneliness among U.S. Army personnel, highlighting the role of organizational-level interventions in mitigating loneliness and related health consequences. The present study focuses specifically on perceived stress as a mechanism that could be the target of future loneliness interventions implemented at either the individual or the organizational level.

As service members are already at a greater risk for psychological distress (Nichter et al., 2020), alcohol misuse (e.g., Bray et al., 2010), and sleep insufficiencies (Farhadian et al., 2022), health and behavioral consequences of loneliness are likely amplified in military samples. Indeed, Na et al. (2022) and Kelly et al. (2022) found that, among other risk factors, loneliness was a significant contributor to the development of alcohol use disorders among veterans during the COVID-19 pandemic. Within a sample of older US veterans, Straus et al. (2022) identified that those reporting feeling lonely “often” were more likely to experience multiple physical health diagnoses, including sleep disorders. Studies by Straus et al. (2022), Na et al. (2022) and Kelly et al. (2022) are seminal in that they generalize known outcomes of loneliness to military samples. However, findings were mostly derived from cross-sectional studies and relied on small and selective samples (e.g., older adult veterans). Thus, conclusions about generalizability and the directionality of interrelationships among these variables cannot be made.

Further, while research on civilian samples has established perceived stress as one mechanism through which loneliness undermines health, little work has examined this process in military samples. Existing research, though limited, reveals moderate correlations between veteran loneliness and perceived stress (J. T. Cacioppo et al., 2016; Kuwert et al., 2014), and that lonely veterans are more susceptible to depression, due to higher levels of stress (J. C. Martin & Hartley, 2017). Notably, these studies assessed global stress, rather than specific dimensions (e.g., perceived helplessness and coping appraisal), which may play unique roles in loneliness–health associations. Understanding the distinct roles of these stress-related mechanisms is crucial for the continued development of effective interventions in both military and civilian contexts.

### 1.5. Present Study

The current study extends previous research by investigating whether loneliness predicts greater stress, poorer sleep, and higher alcohol misuse and psychological distress in a military population. We also test two dimensions of perceived stress—perceived helplessness and negative coping appraisal—as mechanisms linking loneliness to health outcomes, and assess prospective associations, clarifying directionality in loneliness–stress–health pathways. Further, we seek to replicate existing research from nonclinical, community-dwelling adults with a sample of active-duty service members (SMs). Based on previously established links among loneliness, stress, and health more generally, we propose that greater loneliness at baseline will predict greater perceived helplessness and more negative coping appraisals at a 4-month follow-up. We expect that baseline loneliness will subsequently predict poorer sleep, greater alcohol misuse, and greater psychological distress at time two via perceived helplessness and coping appraisals. These associations will occur above and beyond baseline levels of stress, sleep, alcohol use, and psychological stress, thus demonstrating directional consequences of loneliness on stress and health. Results will contribute to the increased understanding of avenues by which loneliness is consequential to health, in the military context.

## 2. Materials and Methods

### 2.1. Participants and Procedure

Data were derived from a larger cluster-randomized control trial testing the efficacy of a supportive-leadership intervention (U.S. Army active-duty platoon leaders) on active-duty SMs’ perceptions of social connection. The focus of the current study is on SM’s self-reported loneliness, stress, and health at baseline and at a 4-month follow-up; as we do not test for group differences in intervention effects, the study condition was included as a control in all analyses. Participants were recruited from a U.S. Army military installation in the Western United States. Eligible SMs were those who were assigned to study battalions who did not identify as platoon leaders. The top three participating platoons received pizza parties for each survey wave, in return for study participation. Baseline and follow-up surveys were completed online, approximately one month before the larger study intervention, and three months following the intervention; respondents were free to skip questions on the survey with no penalty and ensured confidentiality of their responses.

The analysis sample for the current study includes responses from SMs who completed surveys at both time points (baseline, 4-month follow-up). Whereas a total of 1890 surveys were completed by eligible SMs at baseline, only 813 surveys were completed at follow-up. A total of 299 participants completed surveys at both time points.^1^ Data collection occurred soon after the start of the COVID-19 pandemic, with baseline administration occurring across 6 weeks in fall 2020 and follow-up survey administration across 6 weeks in winter 2021. Due to COVID-19 restrictions, all surveys were administered via a remote online data collection protocol. Importantly, all data collection occurred after the start of the pandemic; thus, COVID-19 is considered a constant in our sample.

As we accounted for nesting at the company level (see data analysis plan), 23 participants were excluded from our final sample because of missing company info, thus yielding a total sample size of *N* = 276 for the current analyses; 43% of sample participants had supervisors who were assigned to the supportive-leadership intervention group. The analysis sample was 88.4% men and 8.3% women, and 3.6% did not provide sex. SMs were on average 23.88 years old (SD = 4.68). In terms of race/ethnicity, SMs were asked to select all that apply; 30.4% selected multiple options. Most SMs indicated White (60.5%) as their race, followed by 23.5% Hispanic/Latinx, 13.8% Black, 6.5% Asian, 4.3% Hawaiian/Pacific Islander, and 3.9% Native American or Alaskan Native. Regarding education, 54.8% reported completing high school or less than high school; 28.5% some college or technical school, no degree; 13.3% completed college with a degree or certificate; and 3% completed graduate study.

### 2.2. Measures

Loneliness. Baseline loneliness was measured via the Brief Loneliness Scale (Hughes et al., 2004). Participants responded to three items using a 4-point Likert-type scale (1 = Never, 4 = Always; i.e., “How often do you feel left out?”, “How often do you lack companionship?”, “How often do you feel isolated from others?”). Responses were summed to create a composite loneliness score (alpha = 0.91T1).

Stress. The four-item version of the Perceived Stress Scale (Cohen, 1988) was used to measure past-month stress at baseline and follow-up. Participants were asked to respond to four items using a 5-point Likert-type scale, with reference to the past month (1 = Never, 5 = Very Often; e.g., “How often have you felt that you were unable to control the important things in your life?”). Following recommendations made by Leung et al. (2010), two negatively-worded items were combined to form the perceived helplessness subscale, and two positively-worded items were reverse-scored and combined to form the negative coping appraisal subscale; higher scores on each represent greater perceived helplessness and more negative coping appraisals. Taken together, items on the PSS-4 composite exhibited questionable reliability at both time points (alphas = 0.63T1 and 0.59T2). Reliability improved when scale items were modeled as two factors across both time points (alphas for perceived helplessness items = 0.73T1 and 0.78T2; alphas for negative coping appraisal items = 0.76T1 and 0.83T2). Perceived helplessness (PH) and negative coping appraisal (NCA) subscales were moderately correlated at T1 (r = 0.18, *p* = 0.001) but were not correlated at T2 (r = −0.01, *p* = 0.908).

Alcohol misuse. Self-reported alcohol misuse was assessed at baseline and the 4-month follow-up via the 3-item AUDIT-C (Bush et al., 1998). Participants indicated general frequency of alcohol use (0 = Never, 1 = Monthly or less, 2 = 2 to 3 times a week, 4 = 4 or more times a week), as well as the number of standard drinks consumed on a typical drinking day (0 = 1 or 2 to 4 = 10 or more), and the frequency at which they consumed 6 or more drinks in one drinking occasion (0 = Never to 4 = Daily or almost daily). Items were summed to create a composite alcohol misuse score at each timepoint, with higher scores indicating a greater level of misuse (alphas = 0.72T1 and 0.78T2).

Sleep. Past 30-day sleep disturbance was assessed via the Patient-Reported Outcomes Measurement Information System (PROMIS; Yu et al., 2012); the current analyses were specifically focused on insomnia symptoms and dissatisfaction with sleep. At each time point, participants reported the extent to which they had trouble falling asleep (1 = Not at all, 5 = Very Much; 4-items; e.g., “I had trouble sleeping”., “I got enough sleep”.) and whether they were satisfied with their sleep quality (1 = Never, 5 = Always; 4-items; e.g., “My sleep was refreshing”., “I got enough sleep”.). T-score transformations for each subscale were calculated using the HealthMeasures scoring system. Both subscales demonstrate acceptable reliability at baseline and at follow-up (insomnia symptoms: alphas = 0.89T1, 0.90T2; sleep dissatisfaction: alphas = 0.90T1, 0.91T2).

Psychological Distress. Participants responded to past 30-day emotional distress via the 6-item Kessler Emotional/Psychological Distress Scale (Kessler et al., 2002); e.g., “About how often did you feel so depressed that nothing could cheer you up?”. Items were rated on a 4-point Likert-type scale (1 = None of the time, 4 = Most of the time; alphas = 0.92T1 and 0.93T2) and averaged to create a composite distress score at each timepoint.

### 2.3. Data Analytic Plan

Proposed prospective associations among loneliness at baseline (T1), perceived helplessness (PH), negative coping appraisal (NCA), alcohol misuse, sleep, and psychological distress at 4 months post-baseline (T2) were examined via structural equation modeling in Mplus v. 8 (Muthén & Muthén, 2017). Indirect effects were tested via a series of multiple-mediation models, wherein perceived helplessness (PH) and negative coping appraisal (NCA) were modeled as parallel mediators across loneliness-health outcome models. Continuous predictors were grand-mean centered, and alcohol misuse, insomnia symptoms, sleep dissatisfaction, and psychological distress outcomes were tested in separate models. Results of preliminary ANOVAs revealed significant clustering based on company for alcohol outcomes (F(34, 236) = 1.700, *p* = 0.013) and insomnia symptoms (F(34, 236), *p* < 0.027) at T1. To maintain consistency across models, we accounted for clustering based on company across all outcomes. The treatment group was included as a control variable in all analyses, as intervention effects were not a focus of the current study.

Paths were modeled between loneliness at T1 to PH and NCA at T2 (PH_T2_ and NCA_T2_); PH_T2_ and NCA_T2_ to alcohol misuse, sleep, or psychological distress at T2; and a direct effect between loneliness at T1 and alcohol misuse, sleep, or psychological distress at T2. To control for autoregressive effects, all models adjusted for T1 levels of PH and NCA and corresponding health outcome at T1 (alcohol misuse, insomnia symptoms, sleep dissatisfaction, psychological distress). Indirect effects from loneliness at T1 to alcohol misuse, sleep, and psychological distress at T2, by way of PH_T2_ and NCA_T2_, were estimated via bias-corrected bootstrapped confidence intervals based on 5000 samples.

## 3. Results

**Figure 1:**
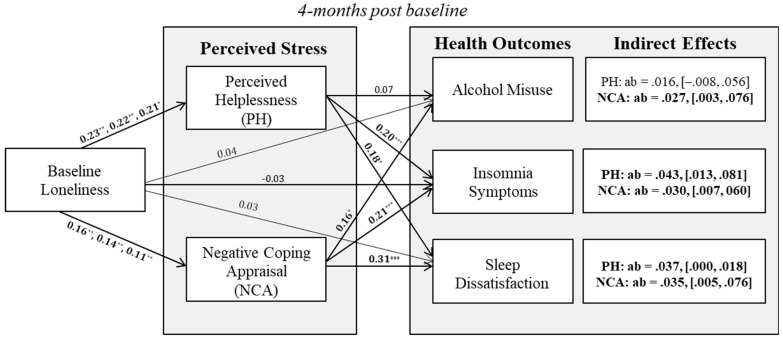
Results of significant indirect effects. For parsimony, baseline values of mediator and outcome variables (control variable) are not shown. For negative coping appraisal, higher scores indicate more negative coping appraisal. There were no significant indirect effects for the psychological distress outcome; thus, model estimates are not displayed in the current figure. Path estimates are standardized coefficients. For the loneliness to mediator paths (path a), estimates from the alcohol misuse model are listed first, followed by estimates from insomnia symptoms, and sleep dissatisfaction models. For indirect effects, *ab* estimates are followed by 95% BC CIs. Slight differences in path estimates for the stress on loneliness association are due to missing data across health outcomes. * *p* < 0.05, ** *p* < 0.01, *** *p* < 0.001.

Descriptive statistics and bivariate correlations are reported in Table 1. Preliminary descriptive analyses revealed associations among age and education with psychological distress and sleep, and gender differences in alcohol misuse. Thus, these variables were considered as covariates in models predicting alcohol misuse, psychological distress, and sleep. As reported in Table 2, controlling for baseline PH and NCA, baseline loneliness significantly predicted greater PH (β = 0.230, *p* = 0.008) and NCA (β = 0.164, *p* = 0.007) at 4 months post-baseline. Subsequently, NCA predicted greater alcohol misuse (β = 0.164, *p* = 0.033), greater insomnia (β = 0.210, *p* < 0.001), and greater sleep dissatisfaction (β = 0.307, *p* < 0.001) at 4 months post baseline, controlling for each respective outcome at baseline. Similarly, perceived helplessness at 4-month post-baseline predicted greater insomnia symptoms (β = 0.196, *p* < 0.001), greater sleep dissatisfaction (β = 0.176, *p* = 0.019), and greater psychological distress (β = 0.274, *p* < 0.001) at follow-up. In models predicting psychological distress, baseline loneliness predicted greater PH (β = 0.174, *p* = 0.048) but not NCA (β = 0.082, *p* = 0.295). Results of tests for multiple mediation (Figure 1; Table 3) revealed significant indirect effects of T1 loneliness on T2 alcohol misuse, insomnia symptoms, and sleep dissatisfaction, via NCA; significant indirect effects emerged for PH on insomnia symptoms and sleep dissatisfaction, but not for alcohol misuse (Figure 1). There were no indirect effects of loneliness on psychological distress, via PH and NCA.

## 4. Discussion

Loneliness has long been understood as a toxic social stressor, activating cognitive and physiological responses that exacerbate disconnection and undermine health over time (J. T. Cacioppo & Cacioppo, 2018; Hawkley et al., 2003). Transactional models of stress and coping (Lazarus & Folkman, 1984) suggest that how individuals appraise their stressors impacts coping effectiveness and subsequently downstream health outcomes, including substance use and sleep. Further, tension reduction (Conger, 1956) and stressor-vulnerability models of drinking behavior (Armeli et al., 2000) suggest that negative emotion and stress are important predictors of alcohol use, though these associations are partially contingent on individual differences in perceived coping resources. Consistent with these perspectives, our findings show that service members who felt lonelier were more likely to perceive themselves as helpless or lacking coping resources, and these appraisals, in turn, predicted greater alcohol misuse and poorer sleep health. By identifying stress appraisals as distinct pathways linking loneliness to both drinking and sleep outcomes, our findings help clarify why loneliness has such broad implications for behavioral health. Importantly, we employed a prospective design to demonstrate that, above and beyond baseline levels of stress and health outcomes, loneliness predicted greater stress and subsequently greater alcohol use and poorer sleep at follow-up. As such, we provide preliminary support for the direction of loneliness, stress, and health associations.

Further, we build on previous research documenting a two-factor model of perceived stress, examining perceived helplessness and negative coping appraisal as parallel mediators and thus highlighting potential targets for intervention. Results of parallel mediator models revealed unique patterns of indirect effects for dimensions of perceived stress on prospective loneliness–health associations. Specifically, negative coping appraisal served as a mechanism in prospective associations among loneliness, alcohol misuse, insomnia, and sleep dissatisfaction. Perceived helplessness helped to explain associations among loneliness, insomnia symptoms, and sleep dissatisfaction, but not alcohol misuse. Interestingly, perceived stress did not explain prospective associations among loneliness and psychological distress (depression). These findings support the two-dimensional model of perceived stress, shedding light on the unique explanatory roles of each dimension of stress for different health outcomes.

Associations between loneliness and health behaviors, specifically substance use, are complex, with some research demonstrating greater alcohol use among the lonely in specific drinking contexts (Arpin et al., 2015) and others demonstrating no differences in use among lonely and non-lonely samples (e.g., Hawkley et al., 2003; Rhew et al., 2021). Previous research has shown that perceived stress more generally explains some of the variability in health behavior among the lonely, with loneliness predicting greater stress and perceived stress predicting greater alcohol and drug use over time (Segrin et al., 2018). Our findings shed new light on mixed findings in previous research concerning health behavior consequences of loneliness, while supporting Segrin et al.’s (2018) findings regarding the explanatory role of perceived stress. That negative coping appraisal (but not perceived helplessness) predicted alcohol misuse follows from the well-supported stressor-vulnerability model of alcohol use (Armeli et al., 2000; Armeli et al., 2007). This model describes stress as a predictor of drinking behavior and ultimately problematic alcohol use. Individuals who expect positive outcomes from drinking (e.g., social enhancement; tension reduction) and those with fewer coping resources or limited coping ability are more likely to drink in response to stress (Cooper et al., 1990). The current study provides initial evidence that loneliness predicts perceived coping deficits, which in turn predict alcohol use. Conversely, perceived helplessness, which was also predicted by loneliness, did not relate to increased drinking behavior at follow-up.

Counterintuitively, the absence of indirect effects for perceived helplessness is also in line with the stressor-vulnerability model, which states that stress-induced drinking is contingent on individual differences such as gender, coping style, alcohol outcome expectancies, and drinking motives (e.g., Cooper et al., 1988; Armeli et al., 2005; Armeli et al., 2007). As a dimension of perceived stress, perceived helplessness in the current study reflects the emotional distress component of stress, specifically perceived lack of control and negative affective reactions, whereas coping appraisal reflects the perceived ability to cope with existing stressors (Leung et al., 2010). Thus, it is possible that associations among perceived helplessness and alcohol misuse in the current study are moderated by individual differences, such as those predicted by the stressor-vulnerability model. Future research should explore potential individual differences in the indirect effects reported here. Of note are drinking motives (e.g., drinking-to-cope) and alcohol expectancies, which previous work has identified as important mechanisms linking negative affect and social stress (i.e., emotional abuse) to alcohol use and alcohol-related problems (Bitsoih et al., 2023). An additional consideration is the role of impaired control (drinking longer or more than intended). Recent research has identified impaired control as an important mediator of the stress–drinking association (Bitsoih et al., 2023; Kalina et al., 2023; Muniz et al., 2024) and an early indicator of alcohol use disorders (Leeman et al., 2012). As loneliness has been shown to undermine self-regulation more generally (Baumeister et al., 2005), investigating impaired control as a mechanism linking loneliness to alcohol use is warranted. Assessing factors that strengthen or buffer the paths to alcohol use identified in the current study would further clarify conditions under which loneliness, via stress, leads to problematic levels of use over time.

The results of the current study also demonstrate the value of exploring different dimensions of sleep health as they relate to loneliness and dimensions of perceived stress. Associations among loneliness and sleep, via stress, are well-established in the previous literature (e.g., McHugh & Lawlor, 2013; Segrin & Burke, 2015). In the current study, we found that some aspects of sleep were more strongly impacted by loneliness-related stress than others. Specifically, negative coping deficits emerged as a stronger predictor of sleep dissatisfaction, while perceived helplessness more strongly predicted insomnia. Kurina et al. (2011) and J. T. Cacioppo and Hawkley (2003) hypothesized that certain experiences of stress cause hypervigilance, resulting in more micro-awakenings and disrupting the soundness of sleep, as is characteristic of insomnia. Preliminary meta-analytic work has shown that the association between stress and sleep is slightly stronger for insomnia symptoms than it is for sleep quality (Gardani et al., 2022). Similarly, a meta-analysis reporting associations among loneliness and sleep found that associations among loneliness and insomnia were slightly stronger than those for loneliness and sleep quality (Griffin et al., 2020). In line with this, Kurina et al. (2011) reported that loneliness predicted sleep fragmentation, but not subjective sleep quality. Our findings build on this work, suggesting that the strength of associations among loneliness, stress, and sleep might depend on how perceived stress is measured. Importantly, in the current study, there were significant indirect effects of perceived helplessness and negative coping appraisal on *both* sleep outcomes, which does suggest some consistency in the impact of stress on sleep. Yet, our findings are preliminary; no other research of which we are aware has differentiated among dimensions of perceived stress in predicting links among loneliness, stress, and sleep health. Future research should seek to replicate our findings with more objective measures of sleep (i.e., actigraphy) that offer more valid and reliable assessments of sleep disruption. Research should also consider the downstream effects of loneliness-related sleep deficits on other health outcomes, including drinking behavior. As insomnia is one mechanism linking social stress (e.g., emotional abuse) to drinking problems (Noudali et al., 2022), it is possible that loneliness, stress, and sleep work together to predict alcohol misuse over time.

Though interesting patterns of indirect effects emerged for alcohol misuse and sleep outcomes, we did not find evidence of indirect effects or perceived stress on psychological distress. The relationship between loneliness and depression is well-known (J. T. Cacioppo et al., 2006b; J. T. Cacioppo et al., 2010), and while the two constructs are strongly related, they are also known to be distinct, with loneliness serving as a risk factor for depression (Hawkley & Cacioppo, 2010; Erzen & Çikrikci, 2018). In the current study, the cross-sectional association between loneliness and psychological distress at baseline was significant, with a moderate to large effect size (r = 0.58, *p* < 0.01). Similarly, the autocorrelation for psychological distress was significant and strong (r = 0.61, *p* < 0.01), providing evidence of stability for this construct over time. The strength of the autocorrelation for psychological distress may have absorbed variance at time two, leaving little residual variance for baseline loneliness or perceived stress to explain (Bollen & Curran, 2006; Curran & Bollen, 2001; Selig & Little, 2012). As such, we were not able to replicate previous research supporting loneliness as a risk factor for depression (Hawkley & Cacioppo, 2010; Erzen & Çikrikci, 2018), as baseline loneliness did not significantly predict follow-up psychological distress (controlling for baseline distress). Future work should consider the combined impact of loneliness and psychological distress on stress, coping appraisals, and subsequent health over time.

### 4.1. Clinical Implications

Findings of the current study are important in that they reveal potential points of intervention for loneliness-related health consequences. Specifically, we show that for different health outcomes, different dimensions of stress may be more or less impactful. That is, loneliness interventions may be more successful when tailored to specific forms of stress and health outcomes. For example, interventions seeking to reduce problematic drinking behavior may be more successful in targeting coping efficacy. Conversely, interventions seeking to improve sleep outcomes among the lonely may do well by targeting feelings of helplessness in addition to coping strategies. Some evidence suggests that mindfulness-based stress reduction (MBSR) is effective in reducing adverse health outcomes of loneliness (Creswell et al., 2012). Future research should build on this work to explore the effects of MBSR on distinct dimensions of stress, which may help to tailor loneliness interventions to specific outcomes of concern. An additional focus of future clinical work should be the consideration of whether known coping responses to loneliness modulate pathways among loneliness, perceived stress, and health. Early loneliness research identified distinct coping strategies that are used in response to loneliness (Rubenstein & Shaver, 1982; Heinrich & Gullone, 2006). These include seeking solitude with the intention of reflecting on and accepting loneliness as an unavoidable part of the human condition (acceptance and resource building); seeking social contact by increasing social participation or soliciting social support from close others (e.g., building social bridges); and distancing and denial, behaviors focused on reducing or numbing social pain, which includes substance use (Rokach & Brock, 1998). Understanding how different loneliness coping strategies relate to or interact with unique dimensions of stress would provide additional insight into how interventions might be tailored to target more specific consequences of loneliness.

### 4.2. Limitations and Future Directions

While our findings significantly contribute to the current literature, results must be interpreted in light of study limitations. Primarily, our assessment of perceived stress relied on the four-item version of the Perceived Stress Scale, which is recommended for long surveys with repeated measures (Demkowicz et al., 2020). The PSS-4 has been shown to have acceptable reliability (Cohen & Williamson, 1988), though longer versions of the scale (PSS-10, PSS-14) are typically recommended when possible (E. H. Lee, 2012). Our decision to use the PSS-4 was due to practical constraints, as the survey captured a host of other measures outside the scope of this paper. Further, given that each dimension of stress was assessed with only two items, the factor structure of the scale could not be confirmed with the current data. As such, we relied on previous evidence supporting its two-factor structure (Taylor, 2015; Demkowicz et al., 2020; Leung et al., 2010). However, in the current study, items on the PSS-4 composite showed questionable reliability at both time points (alphas = 0.63T1 and 0.59T2); reliability improved significantly when scale items were modeled as two factors across both time points (alphas for perceived helplessness items = 0.73 T1 and 0.78 T2; alphas for perceived coping-efficacy items = 0.76 T1 and 0.83T2). These findings are in line with previous research demonstrating poor fit to a single-factor model (Ingram et al., 2016). Future work should seek to replicate our findings with longer versions of the PSS, with the goal of testing the indirect paths explored here and confirming the factor structure of the scale in a military sample.

A related limitation is that we used a unidimensional measure of loneliness. Recent work suggests that loneliness may be a multidimensional construct, which includes feelings of isolation across three primary domains: intimate bonds (intimate/emotional loneliness, or the perceived absence of a significant other), personal/social relationships (relational loneliness, or the perceived absence of quality friendships), and collective space/group memberships (collective loneliness, or the perceived absence of similar others who share some social identity; S. Cacioppo et al., 2015). In the current study, loneliness was measured via the three-item version of the UCLA Loneliness Scale, which has sufficient reliability and is recommended for large survey assessments (Gosling et al., 2024; Hughes et al., 2004). Further, our findings provide important preliminary insight into how loneliness and stress combine to influence health over time in a military sample. However, it is possible that different dimensions of loneliness uniquely impact stress and subsequent health in different contexts, particularly for service members. For example, veterans reintegrating into civilian life may be more likely to experience collective loneliness and thus experience greater stress and poorer health when they feel disconnected from those who share their military social identity. Conversely, service members may experience greater intimate or emotional loneliness during deployment when isolated from intimate partners and family relationships. Walsh et al. (2025) demonstrate how assessing loneliness as a multidimensional versus unidimensional construct provides a clearer understanding of the distinct forms, risks, and implications for loneliness across clinical and public health domains. Further, distinguishing dimensions of loneliness may lead to more precise intervention efforts. As such, exploring how and when service members experience different forms of loneliness (i.e., intimate, personal/social relationships, collective) and the potentially unique downstream consequences on stress and health would be an important and interesting avenue of work to explore.

Lastly, the extent to which these findings can be generalized warrants consideration. While the current study extends previous research on loneliness, stress, and health to a military sample, our sample of service members was limited in diversity in that it was predominantly male (88%) and white (61%). While our sample demographics were not representative of the general population, they were relatively representative of active-duty army units at the time of data collection (68.8% white, AD, all branches; 84.7% male, AD Army; 2019 DoD Demographics report). However, the gender disproportionality in our sample limits generalizability to the general population. Importantly, recent research reveals that the consequences of loneliness are gender-differentiated. For example, whereas women reported greater emotional distress during the pandemic, men were more at risk for increased drinking frequency in response to loneliness, stress, and hopelessness (Thompson et al., 2021; Esper & Furtado, 2013). Similarly, in a study of 17,800 middle-aged adults, loneliness in men was associated with increased alcohol use and a greater likelihood of risky drinking, whereas loneliness predicted reduced risky consumption in women (Johar et al., 2025). Similarly, research supporting the stressor-vulnerability model of alcohol use notes gender as a risk factor for stress-related drinking, with men being at greater risk than women (e.g., Armeli et al., 2000). Yet, recent evidence suggests that women drink more than men in response to social stress (Patock-Peckham et al., 2022) and that the indirect effect of stress on loneliness–drinking associations is stronger for women (Berberian et al., 2022). Relatedly, women experience greater psychological distress in response to social stressors (Kelly et al., 2008) and are more likely to be diagnosed with depression and anxiety-related disorders (Gater et al., 1998).

The sex and gender differences noted above necessitate exploration of gender differences in associations among loneliness, stress, and health. For example, women may exhibit greater perceived helplessness in response to loneliness, and thus greater psychological distress, an indirect effect that did not emerge in the current sample. Recent work by Kalina et al. (2023) reports that, among women, drinking outcomes are more susceptible to the impact of relationship-contingent self-esteem. As a known correlate of self-esteem (Lau et al., 2024), loneliness may similarly make women more susceptible to alcohol use and alcohol-related problems over time. While exploring gender differences would further clarify specific conditions (for whom and when) under which loneliness and stress impact health, the small number of women in our sample limited the power to test for gender differences. Future research should consider whether men and women differ in loneliness-related health outcomes, within a more gender-diverse sample. Amid concern about loneliness as a growing public health threat, this work may shed light on the narrowing gender gap in problematic drinking and alcohol use disorders (Stelander et al., 2021; White, 2020).

An additional study limitation related to generalizability is that data collection occurred during the COVID-19 pandemic, with survey administration occurring in fall 2020 (baseline) and winter 2021 (follow-up). This required a change to our survey administration protocol, which was originally designed to be in-person; due to COVID-19 restrictions, we relied on remote data collection, which was more impersonal and likely impacted participant recruitment and retention. Further, levels of loneliness and stress may have been heightened, and the availability of support and coping resources reduced during the duration of the study. However, experiences of stress such as those experienced during the COVID-19 pandemic are not uncommon among military service members, who, much like first responders, are frequently embedded in high-stress situations and exposed to trauma due to the nature of their work. We encourage future research on servicemember loneliness and health to prioritize gender and racial/ethnic diversity in samples, and to replicate our findings outside of a global pandemic.

## 5. Conclusions

In conclusion, this study shows that loneliness predicts increased stress (perceived helplessness and coping efficacy), which is subsequently consequential to health behavior and sleep health. Importantly, distinct dimensions of perceived stress uniquely predicted alcohol misuse and sleep outcomes. Perceived stress did not appear to play an explanatory role in the loneliness–psychological-distress association over time. Results highlight the roles of perceived helplessness and negative coping appraisal, as dimensions of stress, in explaining how loneliness impacts health over time. Additionally, observed associations among loneliness, negative coping appraisal, and alcohol misuse are in line with the transactional model of stress and the stressor-vulnerability model of alcohol use, which highlight the role of coping appraisals in responses to stress. Importantly, these findings shed light on inconsistent associations between loneliness and alcohol use typically observed in existing research. We show that loneliness indirectly predicts drinking behavior through perceptions of coping resources. Findings contribute to the current understanding of stress-related processes related to loneliness and health, extending research to a military sample and providing insight into potential targets for loneliness and behavioral health interventions.

## Figures and Tables

**Table 1:** Descriptives and Correlations Among Study Variables.

	N	M (SD)	1	2	3	4	5	6	7	8	9	10	11	12	13	14	15	16	17
1. Lonely_T1_	295	6.38 (2.59)	--																
2. PH_T1_	294	2.47 (1.05)	0.51 **	--															
3. NCA_T1_	294	2.52 (1.05)	0.36 **	0.18 **	--														
4. PH_T2_	295	2.48 (1.04)	0.34 **	0.35 **	0.23 **	--													
5. NCA_T2_	295	2.60 (1.06)	0.30 **	0.25 **	0.45 **	−0.00	--												
6. AUDIT_T1_	292	2.03 (2.53)	−0.00	0.07	0.02	0.08	−0.03	--											
7. AUDIT_T2_	294	2.00 (2.71)	0.13 *	0.09	0.06	0.16 **	0.05	0.52 **	--										
8. Insom_T1_	292	50.63 (11.03)	0.29 **	0.42 **	0.24 **	0.22 **	0.22 **	0.07	0.07	--									
9. Insom_T2_	294	50.92 (11.01)	0.30 **	0.40 **	0.23 **	0.36 **	0.29 **	0.06	0.16 **	0.65 **	--								
10. Slp Dis_T1_	292	52.26 (11.13)	0.31 **	0.34 **	0.36 **	0.23 **	0.38 **	0.15 *	0.13 *	0.63 **	0.55 **	--							
11. Slp Dis_T2_	294	52.21 (10.84)	0.30 **	0.30 **	0.29 **	0.29 **	0.48 **	0.09	0.16 **	0.43 **	0.61 **	0.63 **	--						
12. PsyDist_T1_	294	1.76 (0.79)	0.58 **	0.63 **	0.35 **	0.36 **	0.31 **	0.08	0.13 *	0.55 **	0.48 **	0.41 *	0.29 **	--					
13. PsyDist_T2_	295	1.77 (0.87)	0.46 **	0.40 **	0.36 **	0.50 **	0.27 **	0.10	0.18 **	0.45 **	0.49 **	0.36 **	0.42 **	0.61 **	--				
14. IX	273	0.43 (0.50)	0.01	−0.02	0.02	0.03	−0.04	−0.04	−0.02	−0.02	0.00	0.05	0.04	0.04	−0.03	--			
15. Age	288	23.74 (4.65)	0.00	−0.03	−0.10	−0.05	−0.06	0.09	0.07	−0.11	−0.02	−0.04	−0.09	−0.14 *	−0.07	0.13 *	--		
16. Education	292	2.61 (0.92)	−0.10	−0.02	−0.15 **	0.01	−0.10	0.00	0.05	−0.07	−0.04	−0.11	−0.09	−0.13 *	−0.08	0.10	0.51 **	--	
17. Gender	287	0.08 (0.28)	−0.07	0.09	−0.08	−0.01	−0.08	−0.06	−0.14 *	0.08	0.06	−0.02	−0.05	0.08	0.00	0.10	0.05	0.22 **	--

Note: T_1_ = baseline; T_2_ = 4-month follow-up; PH = perceived helplessness; NCA = negative coping appraisal; AUDIT = Alcohol Misuse; Insom = insomnia symptoms (PROMIS); SlpDis = sleep dissatisfaction (PROMIS); PsyDist = Psychological Distress; IX = Condition: 0 = control group, 1 = treatment group; Gender: 0 = Male, 1 = Female; Education: 1 = Less than high school, 2 = High school diploma/GED, 3 = Some college or technical school, no degree, 4 = Completed college or technical school, with a degree/certificate, 5 = Graduate study in progress or completed, e.g., masters, doctorate, MD; * *p* < 0.05, ** *p* < 0.01.

**Table 2:** Multiple-Mediation Model Results for Alcohol Misuse, Sleep, and Psychological Distress.

	Health Outcomes at 4-Month Follow-Up (T2)											
	Alcohol Misuse			Insomnia Symptoms			Sleep Dissatisfaction			Psychological Distress		
	Beta	SE	Est/SE	Beta	SE	Est/SE	Beta	SE	Est/SE	Beta	SE	Est/SE
NCA_T2_ on Loneliness _T1_	0.16 **	**0.06**	**20.68**	0.14 **	**0.06**	**20.40**	0.11 *	**0.05**	**20.19**	0.08	0.08	0.29
NCA_T1_	0.43 ***	**0.07**	**60.38**	0.42 ***	**0.07**	**60.15**	0.38 ***	**0.07**	**50.35**	0.42 ***	**0.06**	**6.79**
Health outcome _T1_	−0.04	0.05	−0.74	0.07	0.09	0.86	0.22 ***	**0.06**	**30.74**	0.15 ^ʈ^	0.08	1.82
Condition	−0.07	0.04	−10.54	−0.06	0.04	−10.38	−0.06	0.04	−10.59	−0.06	0.04	−1.55
Age	0.05	0.08	0.56	0.05	0.08	−0.06	0.04	0.01	0.49	0.05	0.09	0.62
Education	0.00	0.07	0.04	0.01	0.07	0.08	0.02	0.06	0.24	0.01	0.07	0.13
Gender	−0.02	0.03	−0.57	−0.03	0.03	−0.84	−0.02	0.03	−0.82	−0.04	0.03	−1.24
PH_T2_ on Loneliness _T1_	0.23 **	**0.09**	**20.65**	0.22 **	**0.08**	**20.63**	0.21 *	**0.09**	**20.39**	0.17 *	**0.09**	**1.98**
PH_T1_	0.21 *	**0.09**	**20.42**	0.18 *	**0.09**	**20.02**	0.19 *	**0.08**	**20.27**	0.14	0.10	1.40
Health Outcome _T1_	0.04	0.05	0.72	0.09	0.06	10.58	0.11 *	**0.05**	**20.01**	0.16 ^ʈ^	0.08	1.94
Condition	0.02	0.06	0.37	0.02	0.06	0.38	0.02	0.06	0.29	0.02	0.05	0.31
Age	−0.07	0.06	−10.10	−0.06	0.06	−0.95	−0.06	0.06	−10.06	−0.05	0.06	−0.86
Education	0.06	0.06	0.90	0.06	0.06	0.97	0.06	0.06	10.05	0.07	0.06	1.07
Gender	0.04	0.05	0.68	0.02	0.05	0.48	0.03	0.05	0.65	0.02	0.05	0.37
Hlth_T2_ on NCA_T2_(mediator)	0.16 *	**0.08**	**20.13**	0.21 ***	**0.06**	**30.58**	0.31 ***	**0.07**	**40.56**	0.10	0.06	1.56
PH_T2_ (mediator)	0.07	0.06	10.10	0.20 ***	**0.05**	**40.13**	0.18 *	**0.08**	**20.35**	0.27 ***	**0.06**	**4.63**
NCA_T1_	−0.07	0.08	−0.86	−0.06	0.07	−0.78	−0.06	0.08	−0.07	0.16 **	**0.06**	**2.70**
PH_T1_	0.02	0.08	0.20	0.07	0.08	0.86	−0.06	0.06	−0.91	0.02	0.07	0.35
Health Outcome _T1_	0.53 ***	**0.06**	**0.20**	0.55 ***	**0.05**	**10.52**	0.48 ***	**0.05**	**90.79**	0.42 ***	**0.11**	**3.85**
Loneliness _T1_	0.04	0.06	0.74	−0.03	0.08	−0.45	0.03	0.08	0.39	0.00	0.06	0.30
Condition	0.04	0.06	0.67	0.01	0.05	0.25	0.01	0.04	0.22	−0.04	0.04	−0.99
Age	−0.01	0.08	−0.12	0.06	0.05	10.06	−0.08	0.05	−10.60	−0.01	0.05	−0.13
Education	0.02	0.05	0.43	−0.05	0.05	−10.11	−0.01	0.08	−0.10	0.02	0.05	0.32
Gender	−0.11 *	**0.05**	**−20.17**	0.04	0.04	10.10	0.05	0.05	0.91	0.02	0.05	0.43

Note: T1 = Baseline, T2 = 4-month follow-up; NCA = negative coping appraisal, PH = perceived helplessness; Hlth = Health Outcome (T1 = baseline, T2 = 4-month follow-up; continuous predictor variables were grand-mean centered; health outcomes were tested in separate models; Condition: 0 = control group, 1 = treatment group; Gender: 0 = Male, 1 = Female; Education: 1 = Less than high school, 2 = HS diploma/GED, 3 = Some college or technical school, no degree, 4 = Completed college or technical school, with a degree/certificate, 5 = Graduate study in progress or completed, e.g., masters, doctorate, MD; * *p* < 0.05, ** *p* < 0.01, *** *p* < 0.001, ^ʈ^ *p* < 0.10.

**Table 3:** Indirect Effects of Loneliness (T1) on Health Outcomes (T2) via Perceived Helplessness and Negative Coping Appraisal (T2).

	Alcohol Misuse			Insomnia Symptoms			Sleep Dissatisfaction			Psychological Distress		
Indirect Effects	Beta	SE	95% BC CI	ab	SE	95% BC CI	ab	SE	95% BC CI	ab	SE	95% BC CI
Total	**0.043**	**0.21**	**0.008, 0.090**	**0.073**	**0.018**	**0.042, 0.111**	**0.072**	**0.030**	**0.022, 0.135**	**0.056**	**0.026**	**0.007, 0.112**
NCA _T2_	**0.027**	**0.018**	**0.003, 0.076**	**0.030**	**0.014**	**0.007, 0.060**	**0.035**	**0.018**	**0.005, 0.076**	0.008	0.011	−0.004, 0.044
PH _T2_	0.016	0.016	−0.008, 0.056	**0.043**	**0.017**	**0.013, 0.081**	**0.037**	**0.029**	**0.000, 0.108**	0.048	0.030	−0.004, 0.114

Note: All indirect effect models adjust for baseline levels of the mediator and outcome variables, treatment condition, age, education, and gender and account for nesting within company; NCA = negative coping appraisal, PH = perceived helplessness; 95% BC CI = bias-corrected confidence interval, 5000 bootstrap resamples. Bold CIs indicate significant indirect effects.

## Data Availability

IRB and Department of Defense, Human Research Protections Office (HRPO) protocol agreements do not permit the posting of data to repositories. Data are available upon request from Leslie B. Hammer (the fourth author).

## Note

1 To account for potential bias due to attrition, we conducted an exploratory logistic regression predicting the likelihood of completing both baseline and follow-up surveys versus just baseline surveys. Specifically, we predicted a dichotomous variable differentiating those in our analysis sample (1) from participants who only responded to the baseline survey (0), as a function of baseline levels of alcohol misuse, sleep, psychological distress, and loneliness. Logistic regression analyses revealed no significant differences as a function of baseline levels of our study outcome variables (*p* values range = 0.28 to 0.85).

## Author Note

The U.S. Army Medical Research Acquisition Activity, 820 Chandler Street, Fort Detrick MD 21702-5014, is the awarding and administering acquisition office. Opinions, interpretations, conclusions, and recommendations are those of the author and are not necessarily endorsed by the Department of Defense. This work was supported by the Office of the Assistant Secretary of Defense for Health Affairs, through the Psychological Health and Traumatic Brain Injury Research Program under Award No. W81XWH-18-PHTBIRP-R2OE-TRA (PI Hammer). Material has been reviewed by the Walter Reed Army Institute of Research. There is no objection to its presentation and/or publication. The opinions or assertions contained herein are the private views of the author and are not to be construed as official or as reflecting true views of the Department of the Army or the Department of Defense. The investigators have adhered to the policies for protection of human subjects as prescribed in AR 70–25.
